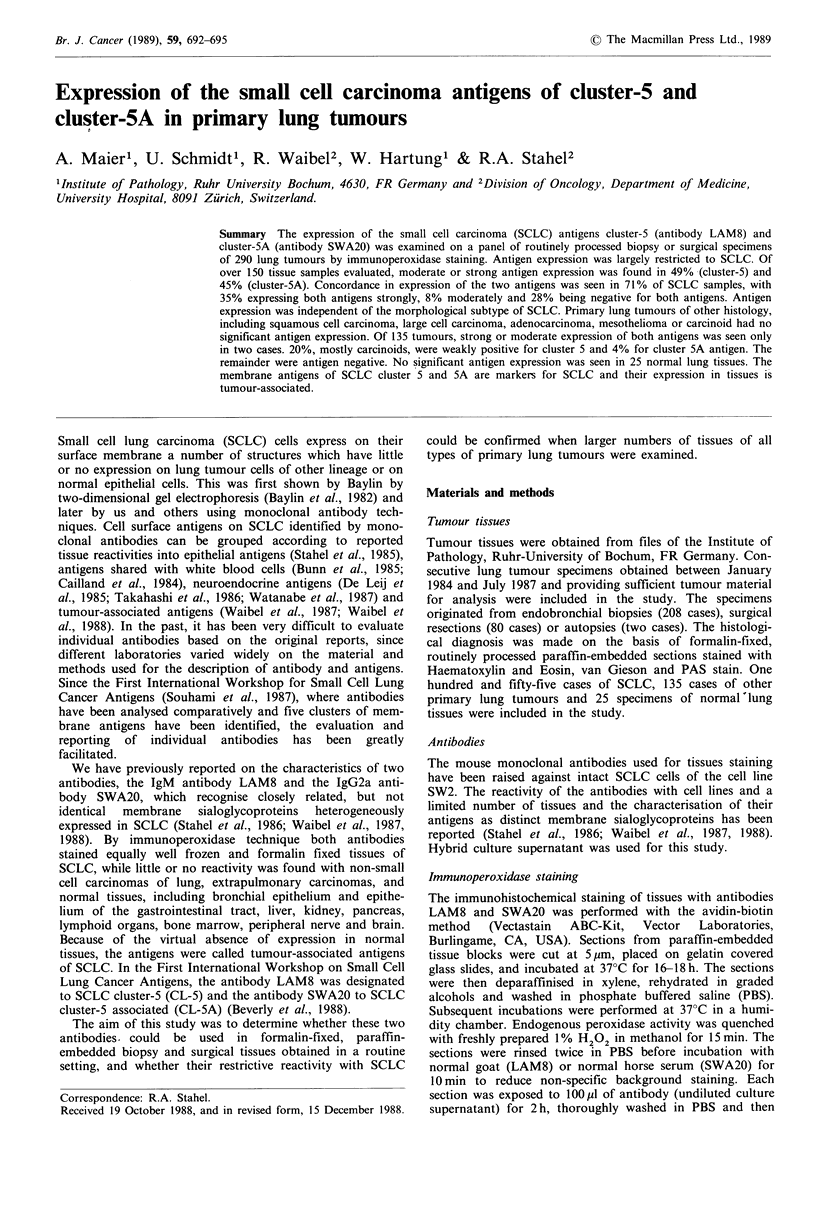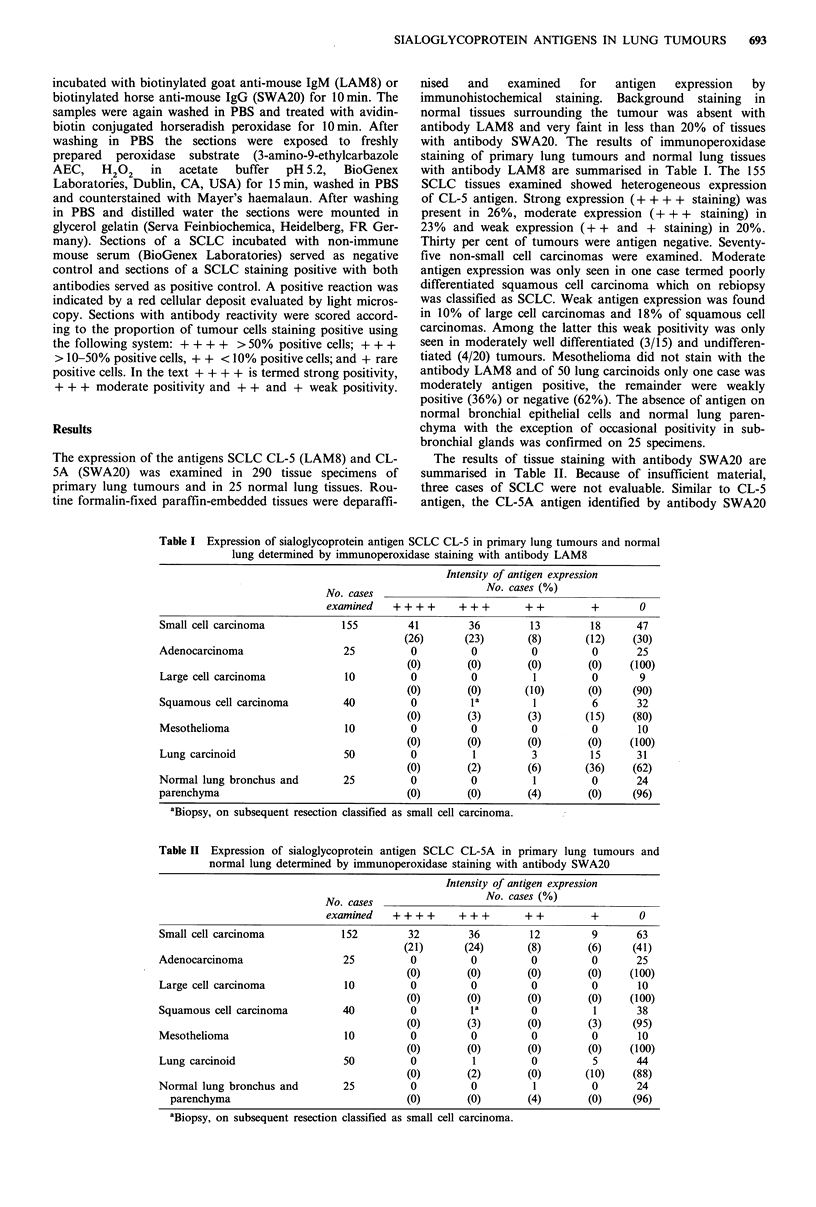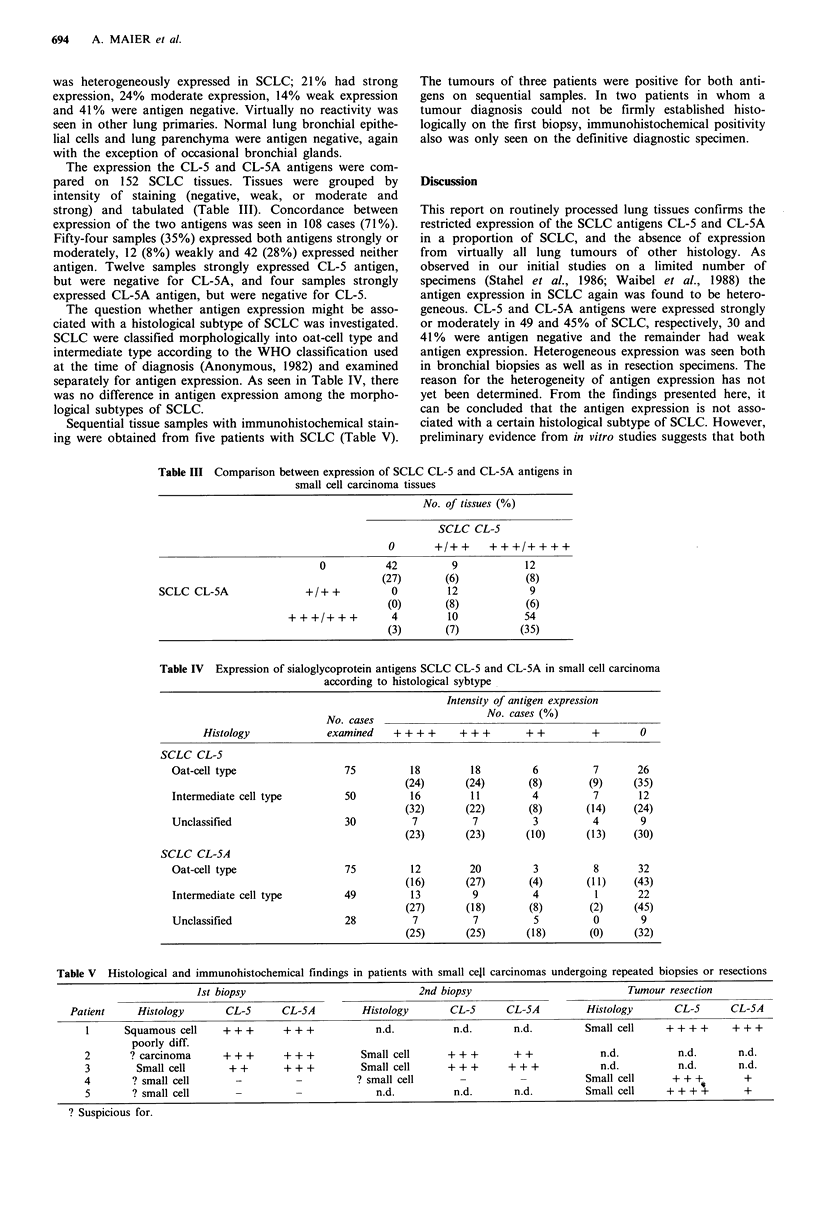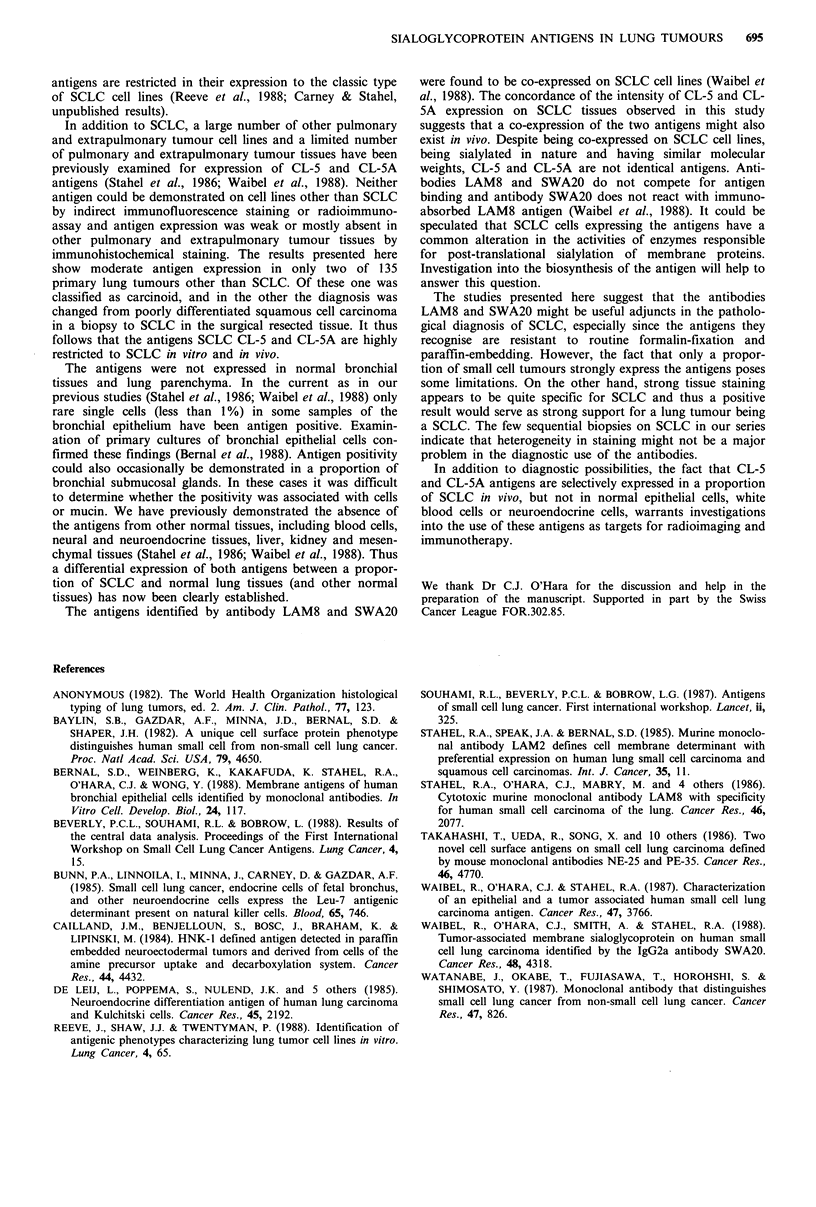# Expression of the small cell carcinoma antigens of cluster-5 and cluster-5A in primary lung tumours.

**DOI:** 10.1038/bjc.1989.144

**Published:** 1989-05

**Authors:** A. Maier, U. Schmidt, R. Waibel, W. Hartung, R. A. Stahel

**Affiliations:** Institute of Pathology, Ruhr University Bochum, FR Germany.

## Abstract

The expression of the small cell carcinoma (SCLC) antigens cluster-5 (antibody LAM8) and cluster-5A (antibody SWA20) was examined on a panel of routinely processed biopsy or surgical specimens of 290 lung tumours by immunoperoxidase staining. Antigen expression was largely restricted to SCLC. Of over 150 tissue samples evaluated, moderate or strong antigen expression was found in 49% (cluster-5) and 45% (cluster-5A). Concordance in expression of the two antigens was seen in 71% of SCLC samples, with 35% expressing both antigens strongly, 8% moderately and 28% being negative for both antigens. Antigen expression was independent of the morphological subtype of SCLC. Primary lung tumours of other histology, including squamous cell carcinoma, large cell carcinoma, adenocarcinoma, mesothelioma or carcinoid had no significant antigen expression. Of 135 tumours, strong or moderate expression of both antigens was seen only in two cases. 20%, mostly carcinoids, were weakly positive for cluster 5 and 4% for cluster 5A antigen. The remainder were antigen negative. No significant antigen expression was seen in 25 normal lung tissues. The membrane antigens of SCLC cluster 5 and 5A are markers for SCLC and their expression in tissues is tumour-associated.


Br. J. Cancer (1989), 59, 692–695 © The Macmillan Press Ltd., 1989

### Expression of the small cell carcinoma antigens of cluster-5 and cluster-5A in primary lung tumours

A. Maier[1], U. Schmidt[1], R. Waibel[2], W. Hartung[1] & R.A. Stahel[2]

[1]*Institute of Pathology, Ruhr University Bochum, 4630, FR Germany and* [2]*Division of Oncology, Department of Medicine, University Hospital, 8091 Zürich, Switzerland.*

**Summary** The expression of the small cell carcinoma (SCLC) antigens cluster-5 (antibody LAM8) and cluster-5A (antibody SWA20) was examined on a panel of routinely processed biopsy or surgical specimens of 290 lung tumours by immunoperoxidase staining. Antigen expression was largely restricted to SCLC. Of over 150 tissue samples evaluated, moderate or strong antigen expression was found in 49% (cluster-5) and 45% (cluster-5A). Concordance in expression of the two antigens was seen in 71% of SCLC samples, with 35% expressing both antigens strongly, 8% moderately and 28% being negative for both antigens. Antigen expression was independent of the morphological subtype of SCLC. Primary lung tumours of other histology, including squamous cell carcinoma, large cell carcinoma, adenocarcinoma, mesothelioma or carcinoid had no significant antigen expression. Of 135 tumours, strong or moderate expression of both antigens was seen only in two cases. 20%, mostly carcinoids, were weakly positive for cluster 5 and 4% for cluster 5A antigen. The remainder were antigen negative. No significant antigen expression was seen in 25 normal lung tissues. The membrane antigens of SCLC cluster 5 and 5A are markers for SCLC and their expression in tissues is tumour-associated.

Small cell lung carcinoma (SCLC) cells express on their surface membrane a number of structures which have little or no expression on lung tumour cells of other lineage or on normal epithelial cells. This was first shown by Baylin by two-dimensional gel electrophoresis (Baylin *et al.*, 1982) and later by us and others using monoclonal antibody techniques. Cell surface antigens on SCLC identified by monoclonal antibodies can be grouped according to reported tissue reactivities into epithelial antigens (Stahel *et al.*, 1985), antigens shared with white blood cells (Bunn *et al.*, 1985; Cailland *et al.*, 1984), neuroendocrine antigens (De Leij *et al.*, 1985; Takahashi *et al.*, 1986; Watanabe *et al.*, 1987) and tumour-associated antigens (Waibel *et al.*, 1987; Waibel *et al.*, 1988). In the past, it has been very difficult to evaluate individual antibodies based on the original reports, since different laboratories varied widely on the material and methods used for the description of antibody and antigens. Since the First International Workshop for Small Cell Lung Cancer Antigens (Souhami *et al.*, 1987), where antibodies have been analysed comparatively and five clusters of membrane antigens have been identified, the evaluation and reporting of individual antibodies has been greatly facilitated.

We have previously reported on the characteristics of two antibodies, the IgM antibody LAM8 and the IgG2a antibody SWA20, which recognise closely related, but not identical membrane sialoglycoproteins heterogeneously expressed in SCLC (Stahel *et al.*, 1986; Waibel *et al.*, 1987, 1988). By immunoperoxidase technique both antibodies stained equally well frozen and formalin fixed tissues of SCLC, while little or no reactivity was found with non-small cell carcinomas of lung, extrapulmonary carcinomas, and normal tissues, including bronchial epithelium and epithelium of the gastrointestinal tract, liver, kidney, pancreas, lymphoid organs, bone marrow, peripheral nerve and brain. Because of the virtual absence of expression in normal tissues, the antigens were called tumour-associated antigens of SCLC. In the First International Workshop on Small Cell Lung Cancer Antigens, the antibody LAM8 was designated to SCLC cluster-5 (CL-5) and the antibody SWA20 to SCLC cluster-5 associated (CL-5A) (Beverly *et al.*, 1988).

The aim of this study was to determine whether these two antibodies could be used in formalin-fixed, paraffin-embedded biopsy and surgical tissues obtained in a routine setting, and whether their restrictive reactivity with SCLC could be confirmed when larger numbers of tissues of all types of primary lung tumours were examined.

Correspondence: R.A. Stahel.
Received 19 October 1988, and in revised form, 15 December 1988.

### Materials and methods

### *Tumour tissues*

Tumour tissues were obtained from files of the Institute of Pathology, Ruhr-University of Bochum, FR Germany. Consecutive lung tumour specimens obtained between January 1984 and July 1987 and providing sufficient tumour material for analysis were included in the study. The specimens originated from endobronchial biopsies (208 cases), surgical resections (80 cases) or autopsies (two cases). The histological diagnosis was made on the basis of formalin-fixed, routinely processed paraffin-embedded sections stained with Haematoxylin and Eosin, van Gieson and PAS stain. One hundred and fifty-five cases of SCLC, 135 cases of other primary lung tumours and 25 specimens of normal lung tissues were included in the study.

### *Antibodies*

The mouse monoclonal antibodies used for tissues staining have been raised against intact SCLC cells of the cell line SW2. The reactivity of the antibodies with cell lines and a limited number of tissues and the characterisation of their antigens as distinct membrane sialoglycoproteins has been reported (Stahel *et al.*, 1986; Waibel *et al.*, 1987, 1988). Hybrid culture supernatant was used for this study.

### *Immunoperoxidase staining*

The immunohistochemical staining of tissues with antibodies LAM8 and SWA20 was performed with the avidin-biotin method (Vectastain ABC-Kit, Vector Laboratories, Burlingame, CA, USA). Sections from paraffin-embedded tissue blocks were cut at 5 $\mu$m, placed on gelatin covered glass slides, and incubated at 37°C for 16–18 h. The sections were then deparaffinised in xylene, rehydrated in graded alcohols and washed in phosphate buffered saline (PBS). Subsequent incubations were performed at 37°C in a humidity chamber. Endogenous peroxidase activity was quenched with freshly prepared 1% $H_2O_2$ in methanol for 15 min. The sections were rinsed twice in PBS before incubation with normal goat (LAM8) or normal horse serum (SWA20) for 10 min to reduce non-specific background staining. Each section was exposed to 100 $\mu$l of antibody (undiluted culture supernatant) for 2 h, thoroughly washed in PBS and then incubated with biotinylated goat anti-mouse IgM (LAM8) or biotinylated horse anti-mouse IgG (SWA20) for 10 min. The samples were again washed in PBS and treated with avidin-biotin conjugated horseradish peroxidase for 10 min. After washing in PBS the sections were exposed to freshly prepared peroxidase substrate (3-amino-9-ethylcarbazole AEC, $H_2O_2$ in acetate buffer pH 5.2, BioGenex Laboratories, Dublin, CA, USA) for 15 min, washed in PBS and counterstained with Mayer's haemalaun. After washing in PBS and distilled water the sections were mounted in glycerol gelatin (Serva Feinbiochemica, Heidelberg, FR Germany). Sections of a SCLC incubated with non-immune mouse serum (BioGenex Laboratories) served as negative control and sections of a SCLC staining positive with both antibodies served as positive control. A positive reaction was indicated by a red cellular deposit evaluated by light microscopy. Sections with antibody reactivity were scored according to the proportion of tumour cells staining positive using the following system: + + + + >50% positive cells; + + + >10–50% positive cells, + + <10% positive cells; and + rare positive cells. In the text + + + + is termed strong positivity, + + + moderate positivity and + + and + weak positivity.

### Results

The expression of the antigens SCLC CL-5 (LAM8) and CL-5A (SWA20) was examined in 290 tissue specimens of primary lung tumours and in 25 normal lung tissues. Routine formalin-fixed paraffin-embedded tissues were deparaffinised and examined for antigen expression by immunohistochemical staining. Background staining in normal tissues surrounding the tumour was absent with antibody LAM8 and very faint in less than 20% of tissues with antibody SWA20. The results of immunoperoxidase staining of primary lung tumours and normal lung tissues with antibody LAM8 are summarised in Table I. The 155 SCLC tissues examined showed heterogeneous expression of CL-5 antigen. Strong expression (+ + + + staining) was present in 26%, moderate expression (+ + + staining) in 23% and weak expression (+ + and + staining) in 20%. Thirty per cent of tumours were antigen negative. Seventy-five non-small cell carcinomas were examined. Moderate antigen expression was only seen in one case termed poorly differentiated squamous cell carcinoma which on rebiopsy was classified as SCLC. Weak antigen expression was found in 10% of large cell carcinomas and 18% of squamous cell carcinomas. Among the latter this weak positivity was only seen in moderately well differentiated (3/15) and undifferentiated (4/20) tumours. Mesothelioma did not stain with the antibody LAM8 and of 50 lung carcinoids only one case was moderately antigen positive, the remainder were weakly positive (36%) or negative (62%). The absence of antigen on normal bronchial epithelial cells and normal lung parenchyma with the exception of occasional positivity in subbronchial glands was confirmed on 25 specimens.

The results of tissue staining with antibody SWA20 are summarised in Table II. Because of insufficient material, three cases of SCLC were not evaluable. Similar to CL-5 antigen, the CL-5A antigen identified by antibody SWA20

**Table I** Expression of sialoglycoprotein antigen SCLC CL-5 in primary lung tumours and normal lung determined by immunoperoxidase staining with antibody LAM8

| | *No. cases examined* | *Intensity of antigen expression No. cases (%)* + + + + | + + + | + + | + | *0* |
|---|---|---|---|---|---|---|
| Small cell carcinoma | 155 | 41 (26) | 36 (23) | 13 (8) | 18 (12) | 47 (30) |
| Adenocarcinoma | 25 | 0 (0) | 0 (0) | 0 (0) | 0 (0) | 25 (100) |
| Large cell carcinoma | 10 | 0 (0) | 0 (0) | 1 (10) | 0 (0) | 9 (90) |
| Squamous cell carcinoma | 40 | 0 (0) | 1[a] (3) | 1 (3) | 6 (15) | 32 (80) |
| Mesothelioma | 10 | 0 (0) | 0 (0) | 0 (0) | 0 (0) | 10 (100) |
| Lung carcinoid | 50 | 0 (0) | 1 (2) | 3 (6) | 15 (36) | 31 (62) |
| Normal lung bronchus and parenchyma | 25 | 0 (0) | 0 (0) | 1 (4) | 0 (0) | 24 (96) |

[a]Biopsy, on subsequent resection classified as small cell carcinoma.

**Table II** Expression of sialoglycoprotein antigen SCLC CL-5A in primary lung tumours and normal lung determined by immunoperoxidase staining with antibody SWA20

| | *No. cases examined* | *Intensity of antigen expression No. cases (%)* + + + + | + + + | + + | + | *0* |
|---|---|---|---|---|---|---|
| Small cell carcinoma | 152 | 32 (21) | 36 (24) | 12 (8) | 9 (6) | 63 (41) |
| Adenocarcinoma | 25 | 0 (0) | 0 (0) | 0 (0) | 0 (0) | 25 (100) |
| Large cell carcinoma | 10 | 0 (0) | 0 (0) | 0 (0) | 0 (0) | 10 (100) |
| Squamous cell carcinoma | 40 | 0 (0) | 1[a] (3) | 0 (0) | 1 (3) | 38 (95) |
| Mesothelioma | 10 | 0 (0) | 0 (0) | 0 (0) | 0 (0) | 10 (100) |
| Lung carcinoid | 50 | 0 (0) | 1 (2) | 0 (0) | 5 (10) | 44 (88) |
| Normal lung bronchus and parenchyma | 25 | 0 (0) | 0 (0) | 1 (4) | 0 (0) | 24 (96) |

[a]Biopsy, on subsequent resection classified as small cell carcinoma.

was heterogeneously expressed in SCLC; 21% had strong expression, 24% moderate expression, 14% weak expression and 41% were antigen negative. Virtually no reactivity was seen in other lung primaries. Normal lung bronchial epithelial cells and lung parenchyma were antigen negative, again with the exception of occasional bronchial glands.

The expression the CL-5 and CL-5A antigens were compared on 152 SCLC tissues. Tissues were grouped by intensity of staining (negative, weak, or moderate and strong) and tabulated (Table III). Concordance between expression of the two antigens was seen in 108 cases (71%). Fifty-four samples (35%) expressed both antigens strongly or moderately, 12 (8%) weakly and 42 (28%) expressed neither antigen. Twelve samples strongly expressed CL-5 antigen, but were negative for CL-5A, and four samples strongly expressed CL-5A antigen, but were negative for CL-5.

The question whether antigen expression might be associated with a histological subtype of SCLC was investigated. SCLC were classified morphologically into oat-cell type and intermediate type according to the WHO classification used at the time of diagnosis (Anonymous, 1982) and examined separately for antigen expression. As seen in Table IV, there was no difference in antigen expression among the morphological subtypes of SCLC.

Sequential tissue samples with immunohistochemical staining were obtained from five patients with SCLC (Table V). The tumours of three patients were positive for both antigens on sequential samples. In two patients in whom a tumour diagnosis could not be firmly established histologically on the first biopsy, immunohistochemical positivity also was only seen on the definitive diagnostic specimen.

### Discussion

This report on routinely processed lung tissues confirms the restricted expression of the SCLC antigens CL-5 and CL-5A in a proportion of SCLC, and the absence of expression from virtually all lung tumours of other histology. As observed in our initial studies on a limited number of specimens (Stahel *et al.*, 1986; Waibel *et al.*, 1988) the antigen expression in SCLC again was found to be heterogeneous. CL-5 and CL-5A antigens were expressed strongly or moderately in 49 and 45% of SCLC, respectively, 30 and 41% were antigen negative and the remainder had weak antigen expression. Heterogeneous expression was seen both in bronchial biopsies as well as in resection specimens. The reason for the heterogeneity of antigen expression has not yet been determined. From the findings presented here, it can be concluded that the antigen expression is not associated with a certain histological subtype of SCLC. However, preliminary evidence from *in vitro* studies suggests that both

**Table III** Comparison between expression of SCLC CL-5 and CL-5A antigens in small cell carcinoma tissues

| | | *No. of tissues* (%) | | |
|---|---|---|---|---|
| | | *SCLC CL-5* | | |
| | | *0* | +/++ | +++/++++ |
| SCLC CL-5A | 0 | 42 (27) | 9 (6) | 12 (8) |
| | +/++ | 0 (0) | 12 (8) | 9 (6) |
| | +++/+++ | 4 (3) | 10 (7) | 54 (35) |

**Table IV** Expression of sialoglycoprotein antigens SCLC CL-5 and CL-5A in small cell carcinoma according to histological sybtype

| *Histology* | *No. cases examined* | *Intensity of antigen expression No. cases* (%) ++++ | +++ | ++ | + | *0* |
|---|---|---|---|---|---|---|
| *SCLC CL-5* | | | | | | |
| Oat-cell type | 75 | 18 (24) | 18 (24) | 6 (8) | 7 (9) | 26 (35) |
| Intermediate cell type | 50 | 16 (32) | 11 (22) | 4 (8) | 7 (14) | 12 (24) |
| Unclassified | 30 | 7 (23) | 7 (23) | 3 (10) | 4 (13) | 9 (30) |
| *SCLC CL-5A* | | | | | | |
| Oat-cell type | 75 | 12 (16) | 20 (27) | 3 (4) | 8 (11) | 32 (43) |
| Intermediate cell type | 49 | 13 (27) | 9 (18) | 4 (8) | 1 (2) | 22 (45) |
| Unclassified | 28 | 7 (25) | 7 (25) | 5 (18) | 0 (0) | 9 (32) |

**Table V** Histological and immunohistochemical findings in patients with small cell carcinomas undergoing repeated biopsies or resections

| | *1st biopsy* | | | *2nd biopsy* | | | *Tumour resection* | | |
|---|---|---|---|---|---|---|---|---|---|
| *Patient* | *Histology* | *CL-5* | *CL-5A* | *Histology* | *CL-5* | *CL-5A* | *Histology* | *CL-5* | *CL-5A* |
| 1 | Squamous cell poorly diff. | +++ | +++ | n.d. | n.d. | n.d. | Small cell | ++++ | +++ |
| 2 | ? carcinoma | +++ | +++ | Small cell | +++ | ++ | n.d. | n.d. | n.d. |
| 3 | Small cell | ++ | +++ | Small cell | +++ | +++ | n.d. | n.d. | n.d. |
| 4 | ? small cell | – | – | ? small cell | – | – | Small cell | +++ | + |
| 5 | ? small cell | – | – | n.d. | n.d. | n.d. | Small cell | ++++ | + |

? Suspicious for.

antigens are restricted in their expression to the classic type of SCLC cell lines (Reeve *et al.*, 1988; Carney & Stahel, unpublished results).

In addition to SCLC, a large number of other pulmonary and extrapulmonary tumour cell lines and a limited number of pulmonary and extrapulmonary tumour tissues have been previously examined for expression of CL-5 and CL-5A antigens (Stahel *et al.*, 1986; Waibel *et al.*, 1988). Neither antigen could be demonstrated on cell lines other than SCLC by indirect immunofluorescence staining or radioimmunoassay and antigen expression was weak or mostly absent in other pulmonary and extrapulmonary tumour tissues by immunohistochemical staining. The results presented here show moderate antigen expression in only two of 135 primary lung tumours other than SCLC. Of these one was classified as carcinoid, and in the other the diagnosis was changed from poorly differentiated squamous cell carcinoma in a biopsy to SCLC in the surgical resected tissue. It thus follows that the antigens SCLC CL-5 and CL-5A are highly restricted to SCLC *in vitro* and *in vivo*.

The antigens were not expressed in normal bronchial tissues and lung parenchyma. In the current as in our previous studies (Stahel *et al.*, 1986; Waibel *et al.*, 1988) only rare single cells (less than 1%) in some samples of the bronchial epithelium have been antigen positive. Examination of primary cultures of bronchial epithelial cells confirmed these findings (Bernal *et al.*, 1988). Antigen positivity could also occasionally be demonstrated in a proportion of bronchial submucosal glands. In these cases it was difficult to determine whether the positivity was associated with cells or mucin. We have previously demonstrated the absence of the antigens from other normal tissues, including blood cells, neural and neuroendocrine tissues, liver, kidney and mesenchymal tissues (Stahel *et al.*, 1986; Waibel *et al.*, 1988). Thus a differential expression of both antigens between a proportion of SCLC and normal lung tissues (and other normal tissues) has now been clearly established.

The antigens identified by antibody LAM8 and SWA20 were found to be co-expressed on SCLC cell lines (Waibel *et al.*, 1988). The concordance of the intensity of CL-5 and CL-5A expression on SCLC tissues observed in this study suggests that a co-expression of the two antigens might also exist *in vivo*. Despite being co-expressed on SCLC cell lines, being sialylated in nature and having similar molecular weights, CL-5 and CL-5A are not identical antigens. Antibodies LAM8 and SWA20 do not compete for antigen binding and antibody SWA20 does not react with immunoabsorbed LAM8 antigen (Waibel *et al.*, 1988). It could be speculated that SCLC cells expressing the antigens have a common alteration in the activities of enzymes responsible for post-translational sialylation of membrane proteins. Investigation into the biosynthesis of the antigen will help to answer this question.

The studies presented here suggest that the antibodies LAM8 and SWA20 might be useful adjuncts in the pathological diagnosis of SCLC, especially since the antigens they recognise are resistant to routine formalin-fixation and paraffin-embedding. However, the fact that only a proportion of small cell tumours strongly express the antigens poses some limitations. On the other hand, strong tissue staining appears to be quite specific for SCLC and thus a positive result would serve as strong support for a lung tumour being a SCLC. The few sequential biopsies on SCLC in our series indicate that heterogeneity in staining might not be a major problem in the diagnostic use of the antibodies.

In addition to diagnostic possibilities, the fact that CL-5 and CL-5A antigens are selectively expressed in a proportion of SCLC *in vivo*, but not in normal epithelial cells, white blood cells or neuroendocrine cells, warrants investigations into the use of these antigens as targets for radioimaging and immunotherapy.

We thank Dr C.J. O'Hara for the discussion and help in the preparation of the manuscript. Supported in part by the Swiss Cancer League FOR.302.85.

### References

ANONYMOUS (1982). The World Health Organization histological typing of lung tumors, ed. 2. *Am. J. Clin. Pathol.*, **77**, 123.

BAYLIN, S.B., GAZDAR, A.F., MINNA, J.D., BERNAL, S.D. & SHAPER, J.H. (1982). A unique cell surface protein phenotype distinguishes human small cell from non-small cell lung cancer. *Proc. Natl Acad. Sci. USA*, **79**, 4650.

BERNAL, S.D., WEINBERG, K., KAKAFUDA, K. STAHEL, R.A., O'HARA, C.J. & WONG, Y. (1988). Membrane antigens of human bronchial epithelial cells identified by monoclonal antibodies. *In Vitro Cell. Develop. Biol.*, **24**, 117.

BEVERLY, P.C.L., SOUHAMI, R.L. & BOBROW, L. (1988). Results of the central data analysis. Proceedings of the First International Workshop on Small Cell Lung Cancer Antigens. *Lung Cancer*, **4**, 15.

BUNN, P.A., LINNOILA, I., MINNA, J., CARNEY, D. & GAZDAR, A.F. (1985). Small cell lung cancer, endocrine cells of fetal bronchus, and other neuroendocrine cells express the Leu-7 antigenic determinant present on natural killer cells. *Blood*, **65**, 746.

CAILLAND, J.M., BENJELLOUN, S., BOSC, J., BRAHAM, K. & LIPINSKI, M. (1984). HNK-1 defined antigen detected in paraffin embedded neuroectodermal tumors and derived from cells of the amine precursor uptake and decarboxylation system. *Cancer Res.*, **44**, 4432.

DE LEIJ, L., POPPEMA, S., NULEND, J.K. and 5 others (1985). Neuroendocrine differentiation antigen of human lung carcinoma and Kulchitski cells. *Cancer Res.*, **45**, 2192.

REEVE, J., SHAW, J.J. & TWENTYMAN, P. (1988). Identification of antigenic phenotypes characterizing lung tumor cell lines *in vitro*. *Lung Cancer*, **4**, 65.

SOUHAMI, R.L., BEVERLY, P.C.L. & BOBROW, L.G. (1987). Antigens of small cell lung cancer. First international workshop. *Lancet*, **ii**, 325.

STAHEL, R.A., SPEAK, J.A. & BERNAL, S.D. (1985). Murine monoclonal antibody LAM2 defines cell membrane determinant with preferential expression on human lung small cell carcinoma and squamous cell carcinomas. *Int. J. Cancer*, **35**, 11.

STAHEL, R.A., O'HARA, C.J., MABRY, M. and 4 others (1986). Cytotoxic murine monoclonal antibody LAM8 with specificity for human small cell carcinoma of the lung. *Cancer Res.*, **46**, 2077.

TAKAHASHI, T., UEDA, R., SONG, X. and 10 others (1986). Two novel cell surface antigens on small cell lung carcinoma defined by mouse monoclonal antibodies NE-25 and PE-35. *Cancer Res.*, **46**, 4770.

WAIBEL, R., O'HARA, C.J. & STAHEL, R.A. (1987). Characterization of an epithelial and a tumor associated human small cell lung carcinoma antigen. *Cancer Res.*, **47**, 3766.

WAIBEL, R., O'HARA, C.J., SMITH, A. & STAHEL, R.A. (1988). Tumor-associated membrane sialoglycoprotein on human small cell lung carcinoma identified by the IgG2a antibody SWA20. *Cancer Res.*, **48**, 4318.

WATANABE, J., OKABE, T., FUJIASAWA, T., HOROHSHI, S. & SHIMOSATO, Y. (1987). Monoclonal antibody that distinguishes small cell lung cancer from non-small cell lung cancer. *Cancer Res.*, **47**, 826.